# Non-aromatic Anticonvulsant (Divalproex Sodium)-Induced Drug Reaction With Eosinophilia and Systemic Symptoms (DRESS) Syndrome

**DOI:** 10.7759/cureus.17860

**Published:** 2021-09-09

**Authors:** Priyata Dutta, Sulagna Das, Adam Fershko

**Affiliations:** 1 Internal Medicine, Mymensingh Medical College, Mymensingh, BGD; 2 Internal Medicine, Kettering Medical Center, Kettering, USA

**Keywords:** dress syndrome, anti-epileptics, skin reaction, liver failure, valproic acid

## Abstract

A wide array of commonly prescribed antiepileptic medications, antibiotics, antivirals, anti-parasitic, and antihyperuricemic can cause Drug Reaction Eosinophilia and Systemic Syndrome (DRESS)- a drug induced hypersensitivity reaction characterized by cutaneous manifestation, fever, eosinophilia, thrombocytopenia and one or more visceral organ involvement. The rare occurrence in clinical settings and wide variety of clinical presentations make DRESS a diagnostically challenging case. A vast majority of DRESS cases are attributed to the most commonly prescribed anticonvulsant medications - phenytoin and carbamazepines. Even though non-aromatic divalproex sodium/valproic acid (VPA) can cause life-threatening fatal hypersensitivity reactions on rare occasions, a handful cases of valproate-induced DRESS have been reported. We hereby report a case of a 57-year-old cognitively impaired person with polypharmacy who presented with worsening diffuse skin rash, fever, dysphagia, eosinophilia, thrombocytopenia, and mixed type of hepatic injury. The patient was eventually diagnosed with DRESS due to divalproex sodium- an anticonvulsant medication. The objective of our report is to identify and recognize the rare yet proper causative agent that induces DRESS and potential mitigation of significant systemic consequences by its subsequent withdrawal.

## Introduction

DRESS is a rare yet potentially fatal condition characterized by morbilliform skin rash, fever, enlarged lymph nodes, systemic involvement of one or more organs, and hematological manifestations like eosinophilia and atypical lymphocytes. This term was first described by Bocquet and his colleagues in 1996. It generally affects 1 in every 1000-10,000 exposures and results in 10-20% of patients dying mostly in fulminant hepatic failure [1-3]. This hypersensitivity reaction occurs due to exposure to a wide range of medications including both aromatic and non-aromatic anticonvulsants, allopurinol, vancomycin, linezolid, sulfonamides, oxicams, minocycline, dapsone, antiretrovirals, and nitrofurantoin [2-5]. However, the pathogenesis is still unknown and probably multifactorial including immunological and genetically associated factors [6]. Very often, an infectious etiology such as viral reactivation also plays a potential role in this clinical entity. Although signs and symptoms develop following the exposure to the culprit drugs, the latency period ranges from two to eight weeks depending on the nature of the drugs, and sometimes it can delay up to more than 100 days. The resolution takes place after the cessation of the causal drugs [7,8].

## Case presentation

A 57-year-old man with a history of intellectual disability and cognitive impairment was admitted to the Emergency Department (ED) from an extended care facility with a one-week history of generalized, morbilliform erythematous rash associated with a fever of 100.3 °F (39.4 °C) and moderate dysphagia followed by eosinophilia, thrombocytopenia, elevated transaminase enzymes and proteus bacteremia of the presumed urinary source. On admission, the patient was extremely agitated and he was unable to provide any history. Hence, it was unsure whether the patient had any associated pruritus or not. According to the ED documentation, the patient received a second dose of the Moderna vaccine the day before admission. 

About two weeks prior, the patient was treated with hydroxyzine pamoate (Vistaril) 50 mg and diphenhydramine 25 mg at an outpatient department for chronic infestation of scabies. The patient had a past medical history of seizures for which he had been taking divalproex sodium (Depakote) 500 mg and levetiracetam (Keppra) 500 mg. We were unable to get any documents for the course of anticonvulsant treatment. 

**Figure 1:**
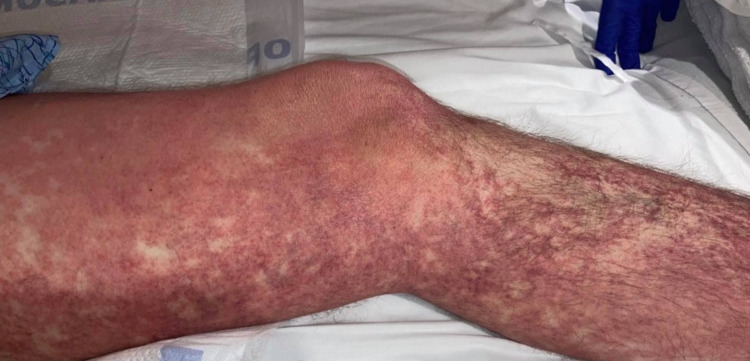
Diffuse morbilliform rash in lower extremity

**Figure 2:**
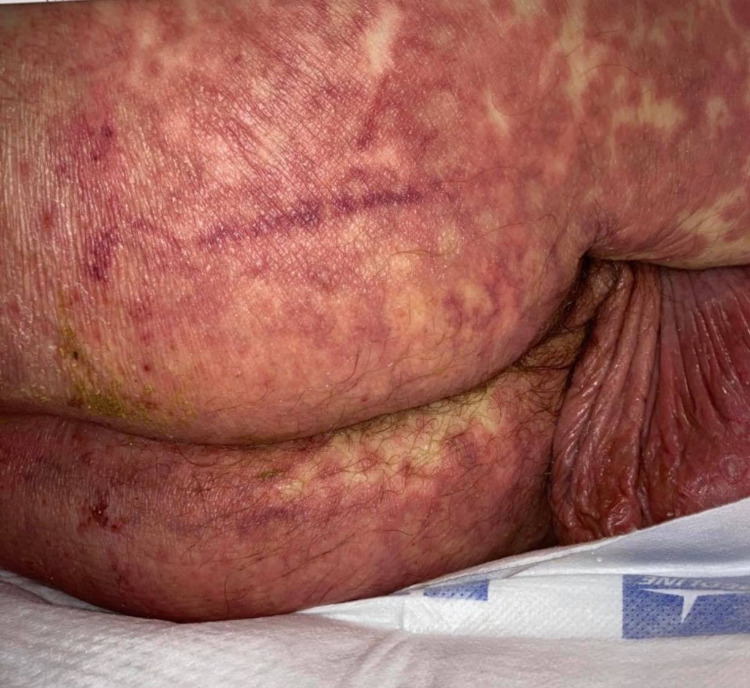
Erythematous patches in the buttock

On physical examination, the patient was alert but febrile, tachycardic, and tachypneic. He had diffuse non-elevated erythematous patches on the face, chest, abdomen, lower extremities, and buttock (Figure 1, 2) without the involvement of oral mucosa, palms, and soles. No vesicles, pustules, bulla, or wheals were noted. On review of the systems, no lymphadenopathy was noted, chest examination showed bilateral clear breath sound without wheezing in auscultation with a normal rate, and regular rhythm of pulse in the cardiovascular examination was found. 

Laboratory results (Table 1) revealed a normal WBC count of 10.4 with a high neutrophil 83.4% and 5.6% eosinophils/ absolute eosinophil .6K/uL (normal 0-3%) followed by 25% after restarting the Depakote, platelet 128,000, hemoglobin 14.1, total bilirubin 4 mg/dl, AST 485 U/L, ALT 657 U/L, ALP 147 U/L, albumin 2.9 g/dl, lactic acid 3.9 mmol/L, Na 148 meq/l, glucose 225 mg/dl, BUN 45 mg/dl, Cr 1.3 mg/dl, urine protein 100 mg/dl, urine nitrite 2+, urine WBC 10-20/HPF, urine bacteria 4+/hpf and blood/urine culture was positive for Proteus. Therefore, the patient was treated with IV Zosyn followed by oral amoxicillin 875 mg for 7 days. The CT abdomen demonstrated mild wall thickening in the urinary bladder with mild hepatomegaly.

**Table 1:** White blood cell count tests in this given patient: *WBC white blood cell count

Laboratory marker	Patient’s value	Ref range
WBC (K/uL)	10.4	4.0-10.5
Eosinophils Percentage	25%	0-3%

Additional work-up with an acute hepatitis panel, HIV, and syphilis were negative. ANA test, Mono test, HHV-6, and HHV-7 were negative as well. The patient was held off all the home medications to identify the causative one and subsequently significant improvement of rash was noted followed by a down-trending of the liver function test (LFT) panel.

On the fourth day, anticonvulsant Depakote was started again and a subsequent increase in transaminase was noted (Table 2). In consensus with the GI consultant, Depakote was discontinued after which LFT began improving. Skin lesions started fading followed by improvement in eosinophilia, and thrombocytopenia. The transaminase continued to improve to 156 and 504 for AST and ALT respectively. At the time of discharge, the patient was on levetiracetam and advised for follow-up with the primary care provider and GI specialist on an outpatient basis. 

**Table 2:** Liver function test panel in the reported patient

	AST (U/L)	ALT (U/L)	ALP (U/L)
Day one	485	657	147
After holding off all home medication	177	451	113
After restarting Depakote	531	942	137
After discontinuation of Depakote	156	504	191

## Discussion

DRESS is an uncommon idiosyncratic reaction that affects mainly adults and is less commonly seen in the pediatric population [9]. It is a type IV hypersensitivity reaction and therefore, like all other hypersensitivity reactions, it causes fever, rash, swelling, eosinophilia, and systemic organ involvement [6, 10]. It is a drug-induced manifestation that can cause a wide range of clinical features, including fever, rash, diffuse swelling, thrombocytopenia, painful peripheral lymphadenopathy, and one or more organ involvement [2,4]. Due to its manifestation of a wide spectrum of clinical features, DRESS is often confused with drug-induced hypersensitivity reactions. The classic triad for DRESS is fever, eosinophilia, and visceral organ involvement [11]. Although Bocquet et al. first presented the diagnostic criteria for DRESS, a widely used scoring criterion by the Registry of Severe Cutaneous Adverse Reaction (RegiSCAR) group is used to diagnose DRESS syndrome (Table 3) [4,12]. In our case, the patient had a high fever, eosinophilia, non-exfoliating rash, thrombocytopenia, hyponatremia, hypoalbuminemia, and mixed type both cholestatic and hepatocellular patterns of liver injury. Our patient scored 6 in the RegiSCAR criteria, consistent with DRESS syndrome.

**Table 3:** The RegiSCAR-Group Diagnosis Score for drug rash with eosinophilia and systemic syndromes (DRESS) *Antinuclear antibody (ANA), serology for Hepatitis A /Hepatitis B/Hepatitis C, chlamydia/mycoplasma, and blood cultures

Symptom	No	Yes	Unknown
Fever (> 38.5C / 101.1F)	-1	0	-1
Enlarged lymph nodes ( > 2 locations, > 1 cm)	0	1	0
Atypical lymphocytes	0	1	0
Eosinophilia: 700-1499 or 10%-19.9% / > 1,500 or > 20%	0	1 / 2	0
Skin rash: Extent >50% / At Least 2 of these: (Edema, Infiltration, Purpura, Scaling )	0 / -1	1 / 1	
Biopsy suggesting DRESS	-1	0	
Internal organ involvement: One / Two or more	0	1 / 2	1
Resolution in 15 days	-1	0	-1
*Biological lab values evaluation: If none positive or >3 negative	0	1	0

Total score 9,

Final score < 2= no case 

Final score 4-5= probable case

Final score >5= definite case.

Regarding the pathogenesis, DRESS is complicated and many factors associated with it are not clearly understood. It has been suggested that hereditary mutation of certain detoxifying enzymes can cause accumulation of toxic drug metabolites, especially some anti-seizure medications that trigger an immunological response - mainly accumulation of interleukin (IL)-5 and eosinophils [13,14]. In addition to this, the association of genetic polymorphism in human leukocyte antigen (HLA) alleles with some particular drugs including aromatic anticonvulsants carbamazepine and anti-inflammatory drug allopurinol may also trigger the DRESS syndrome. Although HLA association with valproic groups has not been demonstrated, genetic predisposition has been considered as one of the potential risk factors for the development of DRESS [13,15]. One recent study proposed that some drugs are implicated for the cytotoxic T cells induced liver injury by cell-contact-dependent mechanisms [16]. However, a clear relationship between viral reactivation and the DRESS syndrome is yet to be established. It has been reported that influenza virus, Epstein-Barr virus (EBV), human herpesviruses 6 and 7 (HHV-6, HHV-7), and cytomegalovirus reactivation can trigger the whole pathophysiology of this disease [14,17,18]. Hence, the aforementioned viral serology is required. For our patient such serology was negative. 

While treating the DRESS, several differential diagnoses should be considered including viral exanthem, primary HIV, steroid-induced drug reaction, Stevens-Johnsons syndrome (SJS), and toxic epidermal necrolysis (TEN) [19]. In our case, the patient didn’t have any vesicles or skin eruption and we excluded other possibilities by a serological test. As our patient also had palpable purpura, we also ruled out the possibility of a disseminated intravascular reaction (DIC), idiopathic thrombocytopenic purpura (ITP), and thrombotic thrombocytopenic purpura (TTP) by normal range of INR, lactate dehydrogenase (LDH), and haptoglobin.

It’s well established that causative drug exposure is the most frequent etiology of DRESS. Several well-known medications including anticonvulsants, antibiotics, antivirals, and most commonly prescribed antihypertensive medications are the most common causative agents. Among anticonvulsants, aromatic groups like phenytoin (5%-7%), carbamazepine (5%-17%), lamotrigine (5%-10%), and phenobarbitone are most frequently associated with drug-induced cutaneous reactions. Very rarely, non-aromatic agents such as valproate, levetiracetam, and vigabatrin with other anticonvulsants can also accelerate the manifestation of DRESS [2,15]. Therefore, in contrast with aromatics, very few cases are reported with valproate-associated DRESS syndrome [3,16,19,20]. Clinical symptoms usually take place 8-10 weeks following the drug usage. However, delay up to 4 months in the appearance of symptoms has also been reported and subsequent withdrawal of the drug, supportive treatment and use of corticosteroid have been reported to cause improved outcomes [7,13]. 

In our case, although the exposure time is unknown, cessation of a causal drug and supportive therapy caused prompt recovery of the patient without any complications. In our case, the rarity of causative agent and identification of that drug was quite difficult and complicated as the patient had been on multiple medications without the knowledge of a definite timeline. Therefore, we stopped all the home medications, monitored the patient, and followed by resuming the suspected drug that helped us to identify the potentially rare culprit drug, which was Depakote (divalproex sodium).

## Conclusions

DRESS is a severe cutaneous and systemic disease with a high morbidity and mortality risk. A careful physical examination and prompt management are imperative to halt the progression of this deadly scenario. Despite a lack of adequate knowledge of the drug exposure period, we were successful in identifying the causative agent by holding off medication. Even though non-aromatic anti-epileptics induced DRESS has been rarely reported, our work underlines the necessity of a proper detailed medication history in rapid identification of the causative drug and mitigation of relevant systemic consequences.

## References

[1] Tas S, Simonart T (2003). Management of drug rash with eosinophilia and systemic symptoms (DRESS syndrome): an update. Dermatology.

[2] Bocquet H, Bagot M, Roujeau J (1996). Drug-induced pseudolymphoma and drug hypersensitivity syndrome (Drug Rash with Eosinophilia and Systemic Symptoms: DRESS). Semin Cutan Med Surg.

[3] Darban M, Bagheri B (2016). Drug reaction with eosinophilia and systemic symptoms induced by valproic acid: a case report. Iran Red Crescent Med J.

[4] Oelze LL, Pillow MT (2013). Phenytoin-induced drug reaction with eosinophilia and systemic symptoms (DRESS) syndrome: a case report from the emergency department. J Emerg Med.

[5] Wolkenstein P, Revuz J (1995). Drug-induced severe skin reactions. Incidence, management and prevention. Drug Saf.

[6] Pereira de Silva N, Piquioni P, Kochen S, Saidon P (2011). Risk factors associated with DRESS syndrome produced by aromatic and non-aromatic antipiletic drugs. Eur J Clin Pharmacol.

[7] James J, Sammour YM, Virata AR, Nordin TA, Dumic I (2018). Drug reaction with eosinophilia and systemic symptoms (DRESS) syndrome secondary to furosemide: case report and review of literature. Am J Case Rep.

[8] Cho YT, Yang CW, Chu CY (2017). Drug reaction with eosinophilia and systemic symptoms (DRESS): An interplay among drugs, viruses, and immune system. Int J Mol Sci.

[9] Prylińska M, Dworakowska-Kicińska M, Krogulska A (2021). Dress syndrome in 7-year-old male child - case report. J Mother Child.

[10] Hall DJ, Fromm JS (2013). Drug reaction with eosinophilia and systemic symptoms syndrome in a patient taking phenytoin and levetiracetam: a case report. J Med Case Rep.

[11] Leblebici F, Soyal Ö, Mutlu NM, Yağmurdur H, Karaca O (2014). Diphenylhydantoin Induced DRESS Syndrome: A Case Report. Turk J Anaesthesiol Reanim.

[12] Pannu AK, Saroch A (2017). Diagnostic criteria for drug rash and eosinophilia with systemic symptoms. J Family Med Prim Care.

[13] Scrace B, Fityan A, Bigham C (2020). Drug reactions with eosinophilia and systemic symptoms. BJA Educ.

[14] Lens S, Crespo G, Carrión JA, Miquel R, Navasa M (2010). Severe acute hepatitis in the dress syndrome: report of two cases. Ann Hepatol.

[15] Wu XT, Hong PW, Suolang DJ, Zhou D, Stefan H (2017). Drug-induced hypersensitivity syndrome caused by valproic acid as a monotherapy for epilepsy: first case report in Asian population. Epilepsy Behav Case Rep.

[16] Dreesman A, Hoorens A, Hachimi-Idrissi S (2010). Multiple organ dysfunction syndrome: infection or hypersensitivity reaction?. Eur J Emerg Med.

[17] Girijala RL, Ramamurthi A, Wright D, Kwak Y, Goldberg LH (2019). DRESS syndrome associated with influenza virus. Proc (Bayl Univ Med Cent).

[18] Cacoub P, Musette P, Descamps V, Meyer O, Speirs C, Finzi L, Roujeau JC (2011). The DRESS syndrome: a literature review. Am J Med.

[19] Arévalo-Lorido JC, Carretero-Gómez J, Bureo-Dacal JC, Montero-Leal C, Bureo-Dacal P (2003). Antiepileptic drug hypersensitivity syndrome in a patient treated with valproate. Br J Clin Pharmacol.

[20] Bota RG, Ligasan AP, Najdowski TG, Novac A (2011). Acute hypersensitivity syndrome caused by valproic acid: a review of the literature and a case report. Perm J.

